# Depression and Nicotine Withdrawal Associations with Combustible and Electronic Cigarette Use

**DOI:** 10.3390/ijerph17249334

**Published:** 2020-12-14

**Authors:** Michele L. Pergadia, John W. Newcomer, David G. Gilbert

**Affiliations:** 1 Department of Psychiatry, Washington University School of Medicine, St. Louis, MO 63110, USA; jnewcomer@thrivingmind.org; 2 Thriving Mind South Florida, Miami, FL 33126, USA; 3 Department of Psychology, Southern Illinois University, Carbondale, IL 62901, USA; dgilbert@siu.edu

**Keywords:** nicotine, withdrawal symptoms, depression

## Abstract

Depression is a risk factor for nicotine use and withdrawal. Population level epidemiologic studies that include users of either combustible or electronic cigarette (NICUSER) could inform interventions to reduce nicotine dependence in vulnerable populations. The current study examined the relationship between depression diagnosis (DEPDX), NICUSER, and lifetime rates of DSM-V nicotine withdrawal (NW) symptoms in a nationally representative sample of US adults (*N* = 979), who answered related questions in surveys administered through GfK’s KnowledgePanel. Over 42% of the sample reported lifetime ever combustible cigarette use, 15.6% electronic-cigarette use, and 45.9% either (NICUSER). Weighted logistic regression analyses (controlling for age and gender) found that DEPDX was associated with 2.3 times increased odds (ratio (OR); 95% Confidence Interval (CI): 1.5–3.5) of being a NICUSER. Regarding risks of NW symptoms among NICUSER, models that additionally controlled for frequency of nicotine use found that DEPDX was significantly associated with increased odds of concentration problems (OR = 2.4; 95% CI: 1.3–4.5) and depressed mood (OR = 2.2; 95% CI: 1.1–4.1) when quitting or cutting down on nicotine use. Results highlight the consistent comorbidity between depression, nicotine use, and symptomatic nicotine withdrawal in a population-based sample of combustible and electronic cigarette users.

## 1. Introduction

Convergent evidence from human and animal models finds that nicotine withdrawal is associated with depressive-like symptoms, and that smokers with a clinical history of depression show more severe symptoms [1]. Epidemiologic and clinical studies also indicate that nicotine withdrawal is associated with depression [2,3,4,5,6]. Given the emergence of electronic (e-)cigarettes [7], it is important to re-evaluate the relation between nicotine withdrawal and depression associated with use of conventional/combustible cigarettes as well as e-cigarettes to further inform the clinical and regulatory understanding of the risks in this evolving area. It is reasonable to expect that e-cigarette use, like combustible cigarettes, will be associated with nicotine withdrawal symptoms. Most e-cigarettes contain nicotine [7], including 99% of the e-cigarettes purchased [8]. In addition, while puffing topography can vary across nicotine containing products, it tends to be more consistent within the same individual, and the average amount of nicotine consumed through electronic cigarettes tends to be within the higher range of that consumed when smoking combustible cigarettes [9].

Studies suggest that the use of e-cigarettes is more likely in individuals with psychiatric illness [10,11,12], and while the relation to the number of cigarettes smoked per day and time to first cigarette has been examined [12], the relation to nicotine withdrawal has not. In the current study, we aimed to examine the relationship between depression, nicotine use, and lifetime rates of DSM-V tobacco withdrawal symptoms [13] in a nationally representative sample of US adults, including combustible and e-cigarette users. 

## 2. Materials and Methods

A cross-sectional survey was conducted in a nationally representative sample of U.S. adults (at least 18 years of age and 52% women, *N* = 1029). The sample was derived from panel participants of GfK’s web-enabled KnowledgePanel^®^, a probability-based panel recruited using both random-digit dial telephone and address-based sampling methodologies [14]. When participants initially enrolled into the panel they completed a demographic survey that informs sampling and weighting for survey studies so that they are representative of the US adult population [14]. Our survey was conducted over a 10-day period in 2016 and had a response rate of 62%. Using the post-stratification weights supplied by GfK in analyses, the survey represented and reflected the adult population as specified by the U.S. Census Bureau’s March 2015 Current Population Survey. Florida Atlantic University’s Human Studies Committee approved the protocol as exempt, protocol # 701721-1. 

Measures used in this study, delivered through the GfK web-based data collection system with KnowledgePanel^®^, included smoking-related variables of interest: (i) lifetime ever use of e-cigarettes (at least once) and combustible cigarettes (100 or more cigarettes), and frequency/quantity used per day; (ii) lifetime DSM-V-based tobacco use dependence symptoms [13], including withdrawal symptoms (each scored dichotomously as “yes” or “no”) were assessed in the context of lifetime ever e-cigarette and/or combustible cigarette use; (iii) existing data previously collected by GfK was also provided, including demographic variables of age and gender, which were included as covariates in multivariate models, and depression (self-reported diagnosis of depression by a doctor or other qualified medical professional).

In the current study, weighted analyses were used to examine the relationship between depression (DEPDX), tobacco/nicotine use, and lifetime rates of DSM-V tobacco withdrawal symptoms in a nationally representative sample of U.S. adults (*N* = 979 with complete data), including lifetime ever combustible or electronic cigarette users (NICUSER; *N* = 449), who answered related questions in a survey administered through GfK’s KnowledgePanel.

SAS was utilized for statistical analyses, which can account for the complex survey design and facilitate inclusion of sampling weights to provide nationally representative estimates. Analyses included estimating prevalence rates and conducting chi-square tests using “proc surveyfreq” to characterize the sample in terms of lifetime rates of use, problematic use, and depression. Risk of withdrawal symptoms in lifetime combustible or e-cigarette users with depression versus no depression was also evaluated using logistic regression (“proc surveylogistic”), with control for frequency of nicotine use per day at peak lifetime use, age, and gender.

## 3. Results

Weighted analyses indicated that 42.1% of the sample reported lifetime ever combustible cigarette use (CCIG) with 28.2% of these reporting dual use. A total of 15.6% of the sample reported lifetime ever electronic (e)-cigarette use (ECIG) with 75.1% of these reporting dual use. Furthermore, 45.9% of the sample reported either CCIG or ECIG (NICUSER), and 12.6% of the sample reported having a diagnosis of depression (DEPDX). 

**Figure 1:**
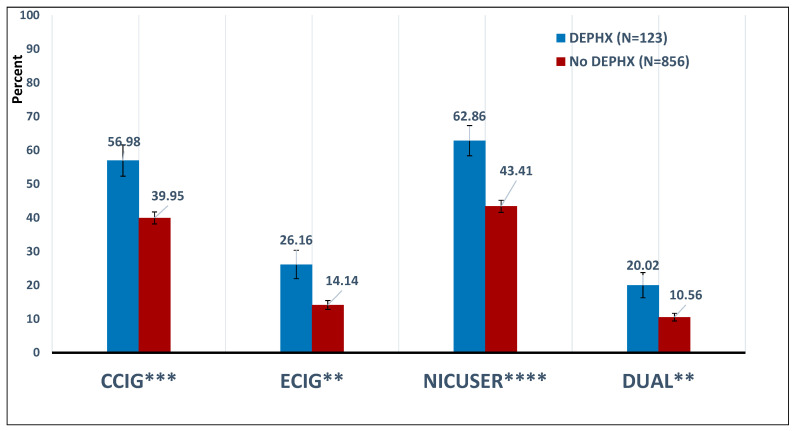
Tobacco Product Use by History of Depression (DEPHX). ** *p* < 0.01; *** *p* < 0.001; **** *p* < 0.0001.

**Figure 2:**
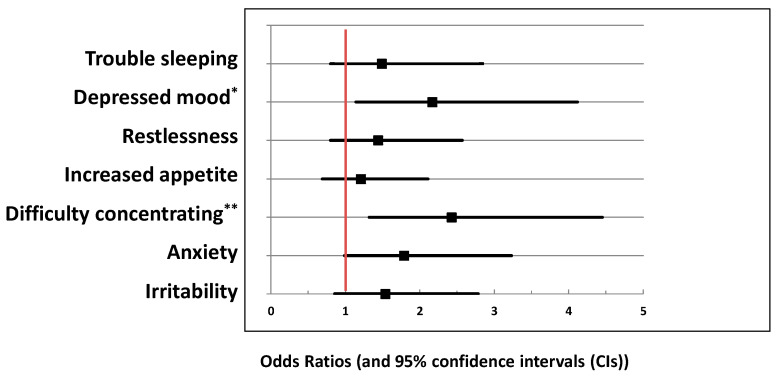
Risk of DSM-V tobacco withdrawal symptoms in electronic- or combustible-cigarette users with depression history compared to no depression history (*N* = 457). * *p* < 0.05; ** *p* < 0.01: results from weighted logistic regression analyses; adjusted odds ratios (and 95% CIs) after controlling for frequency of nicotine use per day, age, and gender; an odds ratio above 1 indicates increased risk of symptom and below 1 decreased risk.

Individuals with depression were significantly more likely to report using CCIG, ECIG, either (NICUSER), or both (DUAL) (all *p*-values were <0.01; see Figure 1). Weighted logistic regression analyses (controlling for age and gender) found that DEPDX was associated with 2.3 times increased odds (ratio (OR); 95% Confidence Interval (CI): 1.5–3.5) of being a NICUSER. Regarding risks of nicotine withdrawal symptoms among NICUSER(s), models that additionally controlled for frequency of nicotine use found that DEPDX was significantly associated with increased odds of concentration problems (OR = 2.4; 95% CI: 1.3–4.5) and depressed mood (OR = 2.2; 95% CI: 1.1–4.1) after quitting or cutting down on cigarette use (see Figure 2).

Weighted logistic regression analyses (controlling for age and gender) examining the risk of withdrawal severity by DEPDX found that DEPDX was significantly associated with an increased risk of one (OR = 2.3; 95% CI: 1.1–4.6), two (OR = 2.1; 95% CI: 1.1–3.8), and all seven (OR = 2.7; 95% CI: 1.1–6.7) symptoms of nicotine withdrawal.

## 4. Discussion

In a nationally representative sample of adults that characterized combustible and e-cigarette use, we found that rates of use were significantly higher in individuals with a history of clinically diagnosed depression. Moreover, we found that depressed users of either combustible or e-cigarettes were significantly more likely to experience nicotine withdrawal symptoms of concentration problems and depressed mood. Those with depression were also significantly more likely to report one to two or all seven nicotine withdrawal symptoms.

While increased use of combustible cigarettes in individuals with depression has been known for some time [15], like other recent work, our results highlight that rates of e-cigarette use are also higher in individuals with depression [10,11,12]. Our work goes further to uniquely show that in a sample with both combustible and e-cigarette users that symptomatic nicotine withdrawal was also more likely in those with depression, particularly concentration problems and depressed mood, along with a greater likelihood of more severe withdrawal as indicated by multiple withdrawal symptoms. These results are highly consistent with previous studies of combustible cigarette use, which also found that depression was specifically associated with increased reports of nicotine withdrawal-related concentration problems and depressed mood, in addition to nicotine withdrawal severity [2,3]. Nicotine abstinence may be more likely to trigger these negative affective symptoms in smokers that have a history of experiencing them in the context of a mood disorder. The similarity in risk for more symptomatic withdrawal and the similar association with depression is not consistent with the idea that e-cigarettes represent a lower-risk smoking alternative.

This study was limited by its cross-sectional nature, and future longitudinal studies are needed to further validate these findings. Although most e-cigarette users are dual users (75% in our sample), future studies should plan to enrich for e-cigarette only users in order to track the unique trends and trajectories of this sub-group.

Taken together, the results highlight the persistent comorbidity between depression, tobacco use, and nicotine withdrawal in a population-based sample with both combustible and electronic cigarette users. Depressed mood and concentration problems, in addition to severe nicotine withdrawal, may be specific targets to consider during cessation treatment in smokers with depression and show consistency across previous studies and this current population-based survey. This study extends that work by examining this relationship in a population study of both combustible and electronic cigarette users. As tobacco use behavior evolves, including the present day epidemic increase in electronic cigarette use [7], strong translational models are critically needed to better understand clinical problems associated with nicotine use.

## 5. Conclusions

In a representative sample, the odds of combustible or electronic cigarette use were more than doubled in individuals with depression. Depression was also associated with increased odds of nicotine withdrawal symptoms, including concentration problems and depressed mood. These results reflect the consistent comorbidity between depression, nicotine use, and symptomatic nicotine withdrawal.
